# Differential attainment in assessment of postgraduate surgical trainees: a scoping review

**DOI:** 10.1186/s12909-024-05580-2

**Published:** 2024-05-30

**Authors:** Rebecca L. Jones, Suwimol Prusmetikul, Sarah Whitehorn

**Affiliations:** 1 Department of Surgery and Cancer, Imperial College London, London, UK; 2 Department of Ophthalmology, Cheltenham General Hospital, Gloucestershire Hospitals NHS Foundation Trust, Alexandra House, Sandford Road, Cheltenham, GL53 7AN UK; 3 Department of Orthopaedics, Faculty of Medicine, Ramathibodi Hospital, Mahidol University, Bangkok, Thailand

**Keywords:** Inequality, Differential attainment, Disparity, Assessment, Diversity, Equity, Postgraduate

## Abstract

**Introduction:**

Solving disparities in assessments is crucial to a successful surgical training programme. The first step in levelling these inequalities is recognising in what contexts they occur, and what protected characteristics are potentially implicated.

**Methods:**

This scoping review was based on Arksey & O’Malley’s guiding principles. OVID and Embase were used to identify articles, which were then screened by three reviewers.

**Results:**

From an initial 358 articles, 53 reported on the presence of differential attainment in postgraduate surgical assessments. The majority were quantitative studies (77.4%), using retrospective designs. 11.3% were qualitative. Differential attainment affects a varied range of protected characteristics. The characteristics most likely to be investigated were gender (85%), ethnicity (37%) and socioeconomic background (7.5%). Evidence of inequalities are present in many types of assessment, including: academic achievements, assessments of progression in training, workplace-based assessments, logs of surgical experience and tests of technical skills.

**Conclusion:**

Attainment gaps have been demonstrated in many types of assessment, including supposedly “objective” written assessments and at revalidation. Further research is necessary to delineate the most effective methods to eliminate bias in higher surgical training. Surgical curriculum providers should be informed by the available literature on inequalities in surgical training, as well as other neighbouring specialties such as medicine or general practice, when designing assessments and considering how to mitigate for potential causes of differential attainment.

**Supplementary Information:**

The online version contains supplementary material available at 10.1186/s12909-024-05580-2.

## Introduction

Diversity in the surgical workforce has been a hot topic for the last 10 years, increasing in traction following the BlackLivesMatter movement in 2016 [1]. In the UK this culminated in publication of the Kennedy report in 2021 [2]. Before this the focus was principally on gender imbalance in surgery, with the 2010 Surgical Workforce report only reporting gender percentages by speciality, with no comment on racial profile, sexuality distribution, disability occurrence, or socioeconomic background [3].

Gender is not the only protected characteristic deserving of equity in surgery; many groups find themselves at a disadvantage during postgraduate surgical examinations [4] and at revalidation [5]. This phenomenon is termed ‘differential attainment’ (DA), in which disparities in educational outcomes, progression rates, or achievements between groups with protected characteristics occur [4]. This may be due to the assessors’ subconscious bias, or a deficit in training and education before assessment.

One of the four pillars of medical ethics is “justice”, emphasising that healthcare should be provided in a fair, equitable, and ethical manner, benefiting all individuals and promoting the well-being of society as a whole. This applies not only to our patients but also to our colleagues; training should be provided in a fair, equitable, and ethical manner, benefiting all. By applying the principle of justice to surgical trainees, we can create an environment that is supportive, inclusive, and conducive to professional growth and well-being.

A diverse consultant body is crucial for providing high-quality healthcare to a diverse patient population. It has been shown that patients are happier when cared for by a doctor with the same ethnic background [6]. Takeshita et al. [6] proposed this is due to a greater likelihood of mutual understanding of cultural values, beliefs, and preferences and is therefore more likely to cultivate a trusting relationship, leading to accurate diagnosis, treatment adherence and improved patient understanding. As such, ensuring that all trainees are justly educated and assessed throughout their training may contribute to improving patient care by diversifying the consultant body.

Surgery is well known to have its own specific culture, language, and social rules which are unique even within the world of medicine [7, 8]. Through training, graduates develop into surgeons, distinct from other physicians and practitioners [9]. As such, research conducted in other medical domains is not automatically applicable to surgery, and behavioural interventions focused on reducing or eliminating bias in training need to be tailored specifically to surgical settings.

Consequently, it’s important that the surgical community asks the questions:

1. Does DA exist in postgraduate surgical training, and to what extent?
2. Why does DA occur?
3. What groups or assessments are under-researched?
4. How can we apply this knowledge, or acquire new knowledge, to provide equity for trainees?

The following scoping review hopes to provide the surgical community with robust answers for future of surgical training.

## Methods

### Aims and research question

The aim of this scoping review is to understand the breadth of research about the presence of DA in postgraduate surgical education and to determine themes pertaining to causes of inequalities. A scoping review was chosen to provide a means to map the available literature, including published peer-reviewed primary research and grey literature.

Following the methodological framework set out by Arksey and O’Malley [10], our research was intended to characterise the literature addressing DA in HST, including Ophthalmology, Obstetrics & Gynaecology (O&G). We included literature from English-language speaking countries, including the UK and USA.

### Search strategy

We used search terms tailored to our target population characteristics (e.g., gender, ethnicity), concept (i.e., DA) and context (i.e., assessment in postgraduate surgical education). Medline and Embase were searched with the assistance of a research librarian, with addition of synonyms. This was conducted in May 2023, and was exported to Microsoft Excel for further review. The reference lists of included articles were also searched to find any relevant data sources that had yet to be considered. In addition, to identify grey literature, a search was performed for the term “differential attainment” and “disparity” on the relevant stakeholders’ websites (See supplemental Table 1 for full listing). Stakeholders were included on the basis of their involvement in governance or training of surgical trainees.

### Study selection

**Fig. 1:**
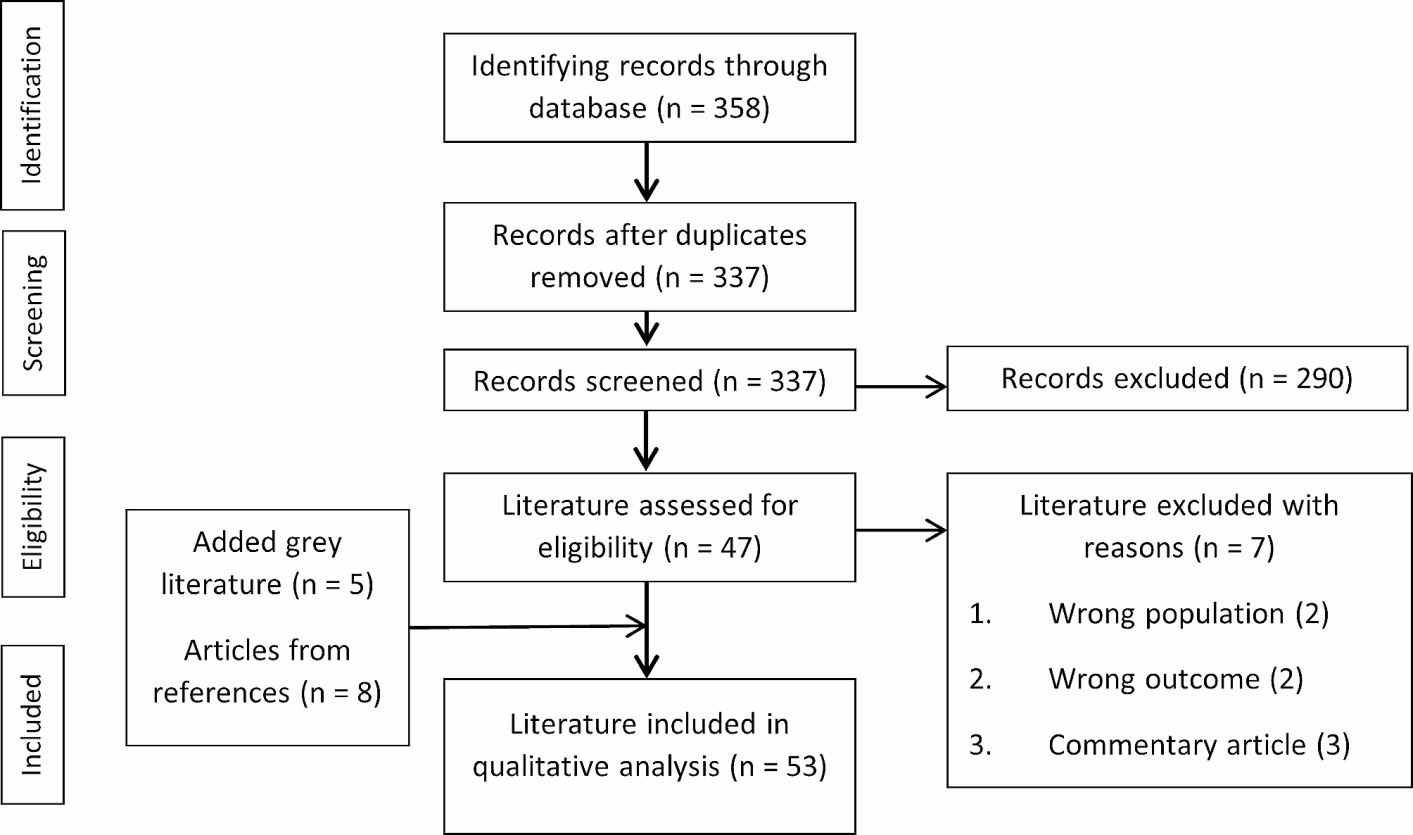
PRISMA flow diagram

To start we excluded conference abstracts that were subsequently published as full papers to avoid duplications (*n* = 337). After an initial screen by title to exclude obviously irrelevant articles, articles were filtered to meet our inclusion and exclusion criteria (Table 1). The remaining articles (*n* = 47) were then reviewed in their entirety, with the addition of five reports found in grey literature. Following the screening process, 45 studies were recruited for scoping review (Fig. 1).

**Table 1:** Inclusion and Exclusion Criteria

Inclusion criteria	Exclusion criteria
Postgraduate training	Recruitment/Applications
Surgical education	Disciplines outside of surgery
English language	Medical student/undergraduate education
Surgical specialties, including Obstetrics & Gynaecology and Ophthalmology	
Focus on assessment	

### Charting the data

The extracted data included literature title, authors, year of publication, country of study, study design, population characteristic, case number, context, type of assessment, research question and main findings (Appendix 1). Extraction was performed initially by a single author and then subsequently by a second author to ensure thorough review. Group discussion was conducted in case of any disagreements. As charting occurred, papers were discovered within reference lists of included studies which were eligible for inclusion; these were assimilated into the data charting table and included in the data extraction (*n* = 8).

### Collating, summarizing and reporting the results

The included studies were not formally assessed in their quality or risk of bias, consistent with a scoping review approach [10]. However, group discussion was conducted during charting to aid argumentation and identify themes and trends.

We conducted a descriptive numerical summary to describe the characteristics of included studies. Then thematic analysis was implemented to examine key details and organise the attainment quality and population characteristics based on their description. The coding of themes was an iterative process and involved discussion between authors, to identify and refine codes to group into themes.

We categorised the main themes as gender, ethnicity, country of graduation, individual and family background in education, socioeconomic background, age, and disability. The number of articles in each theme is demonstrated in Table 2. Data was reviewed and organised into subtopics based on assessment types included: academic achievement (e.g., MRCS, FRCS), assessments for progression (e.g., ARCP), workplace-based assessment (e.g., EPA, feedback), surgical experience (e.g., case volume), and technical skills (e.g., visuo-spatial tasks).

## Results

44 articles defined the number of included participants (89,399 participants in total; range of participants across individual studies 16–34,755). Two articles reported the number of included studies for their meta-analysis (18 and 63 included articles respectively). Two reports from grey literature did not define the number of participants they included in their analysis. The characteristics of the included articles are displayed in Table 2.

**Table 2:** Summary of Characteristics of Identified Articles on DA (*N* = 53)

Domain	Study characteristics	Number (%) of papers (*N* = 53)
Country of origin	United States of America	32 (60.4%)
	United Kingdom	15 (28.3%)
	Canada	1 (1.8%)
	Denmark	3 (5.6%)
	Norway	1 (1.9%)
	Both Ireland and Germany	1 (1.9%)
Types of articles	Quantitative studies	41 (77.4%)
	Retrospective cohort	24
	Prospective cohort	10
	Cross-sectional	3
	Prospective randomised	4
	Qualitative studies	6 (11.3%)
	Interview	2
	Survey	2
	Analysis of written feedback in training reports	2
	Mixed methods	2 (3.8%)
	Review	4 (7.5%)
Study population	General surgery	16 (30.2%)
	Surgical trainees (unspecified specialty)	10 (18.9%)
	Obstetrics & Gynaecology	5 (9.4%)
	Orthopaedics	4 (7.5%)
	Ophthalmology	2 (3.8%)
	Cardiothoracic	2 (3.8%)
	Plastic surgery	1 (1.9%)
	Urology	1 (1.9%)
	Multiple surgical specialties	7 (13.2%)
	Surgical and non-surgical specialties	2 (3.8%)
	Medical students and surgical trainees	3 (5.6%)
Focused characteristic (some articles consider multiple)	Gender	45 (84.9%)
	Ethnicity or country of graduation	16 (37.2%)
	Socioeconomic background	4 (7.5%)
	Individual and family background in education	3 (5.6%)
	Age	4 (7.5%)
	Presence of disability	1 (1.9%)
Type of Assessment (some articles consider multiple)	Academic achievement	18 (34%)
	MRCS	7
	MRCOG	1
	FRCS	2
	ABSCE	3
	OITE	2
	CREOG In-Service Training Examination	1
	ABOS	2
	Assessment for progression	15 (28.3%%)
	ARCP	10
	ACGME Milestone evaluations	5
	Workplace-based assessment	12 (22.6%)
	EPA	2
	Feedback	3
	Specific assessments of clinical performance	1
	ITER	1
	Level of autonomy	5
	Surgical experience	4 (7.5%)
	Number of cases	4
	Technical skills	16 (30.2%)

**Fig. 2:**
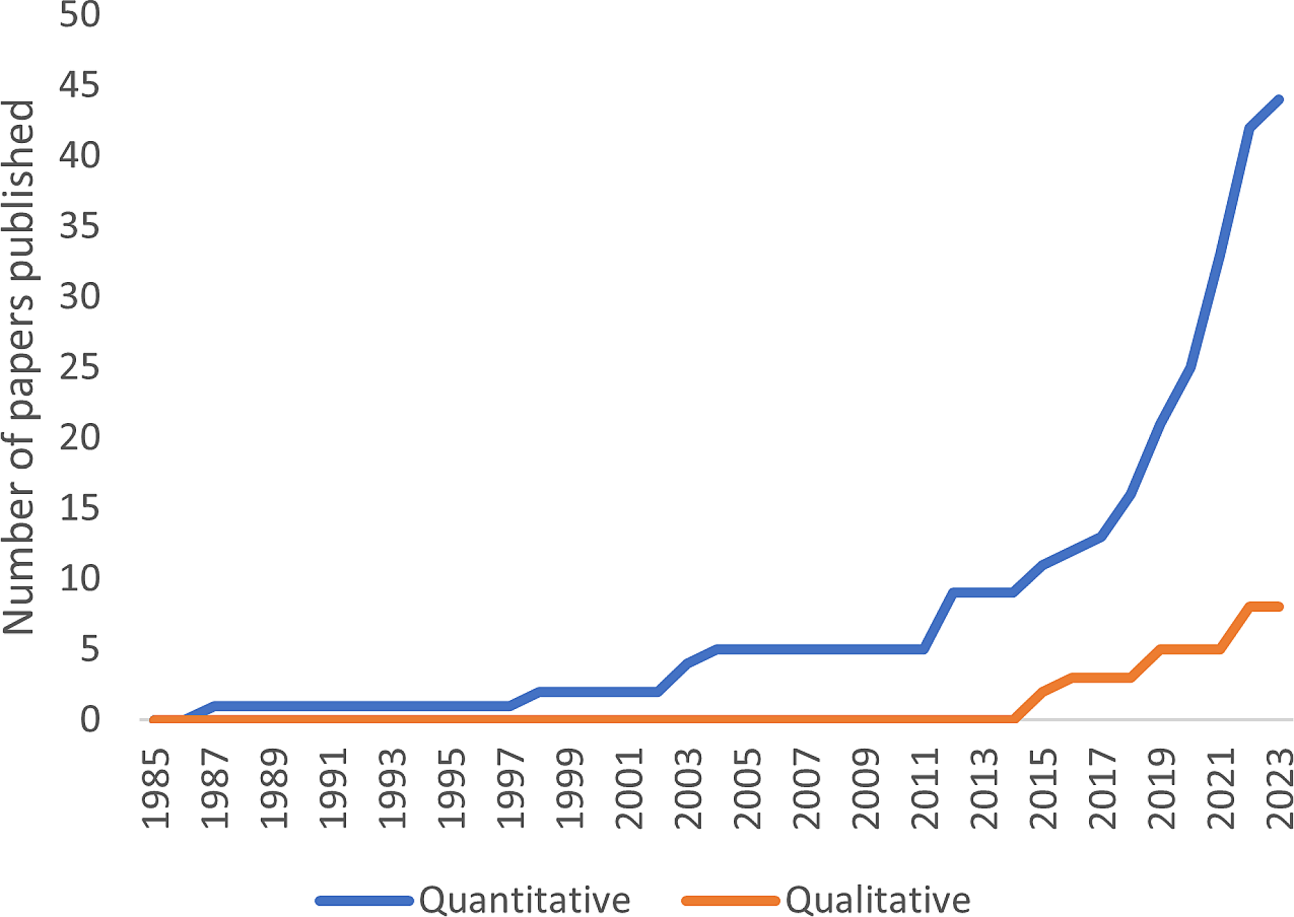
Growth in published literature on differential attainment over the past 40 years

### Gender

#### Academic achievement

In the American Board of Surgery Certifying Exam (ABSCE), Maker [11] found there to be no significant differences in terms of gender when comparing those who passed on their first attempt and those who did not in general surgery training, a finding supported by Ong et al. [12]. Pico et al. [13] reported that in Orthopaedic training, Orthopaedic In-Training Examination (OITE) and American Board of Orthopaedic Surgery (ABOS) Part 1 scores were similar between genders, but that female trainees took more attempts in order to pass. In the UK, two studies reported significantly lower Membership of the Royal College of Surgeons (MRCS) pass rates for female trainees compared to males [4, 14]. However, Robinson et al. [15] presented no significant gender differences in MRCS success rates. A study assessing Fellowship of the Royal College of Surgeons (FRCS) examination results found no significant gender disparities in pass rates [16]. In MRCOG examination, no significant gender differences were found in Part 1 scores, but women had higher pass rates and scores in Part 2 [17].

#### Assessment for Progression

ARCP is the annual process of revalidation that UK doctors must perform to progress through training. A satisfactory progress outcome (“outcome 1”) allows trainees to advance through to the next training year, whereas non-satisfactory outcomes (“2–5”) suggest inadequate progress and recommends solutions, such as further time in training or being released from the training programme. Two studies reported that women received 60% more non-satisfactory outcomes than men [16, 18]. In contrast, in O&G men had higher non-satisfactory ARCP outcomes without explicit reasons for this given [19].

Regarding Milestone evaluations based from the US Accreditation Council for Graduate Medical Education (ACGME), Anderson et al. [20] reported men had higher ratings of knowledge of diseases at postgraduate year 5 (PGY-5), while women had lower mean score achievements. This was similar to another study finding that men and women had similar competencies at PGY-1 to 3, and that it was only at PGY-5 that women were evaluated lower than men [21]. However, Kwasny et al. [22] found no difference in trainers’ ratings between genders, but women self-rated themselves lower. Salles et al. [23] demonstrated significant improvement in scoring in women following a value-affirmation intervention, while this intervention did not affect men.

#### Workplace-based Assessment

Galvin et al. [24] reported better evaluation scores from nurses for PGY-2 male trainees, while females received fewer positive and more negative comments. Gerull et al. [25] demonstrated men received compliments with superlatives or standout words, whereas women were more likely to receive compliments with mitigating phrases (e.g., excellent vs. quite competent).

Hayward et al. [26] investigated assessment of attributes of clinical performance (ethics, judgement, technical skills, knowledge and interpersonal skills) and found similar scoring between genders.

Several authors have studied autonomy given to trainees in theatre [27–31]. Two groups found no difference in level of granted autonomy between genders but that women rated lower perceived autonomy on self-evaluation [27, 28]. Other studies found that assessors consistently gave female trainees lower autonomy ratings, but only in one paper was this replicated in lower performance scores [29–31].

Padilla et al. [32] reported no difference in entrustable professional activity assessment (EPA) levels between genders, yet women rated themselves much lower, which they regarded as evidence of imposter syndrome amongst female trainees. Cooney et al. [33] found that male trainers scored EPAs for women significantly lower than men, while female trainers rated both genders similarly. Conversely, Roshan et al. [34] found that male assessors were more positive in feedback comments to female trainees than male trainees, whereas they also found that comments from female assessors were comparable for each gender.

#### Surgical Experience

Gong et al. [35] found significantly fewer cataract operations were performed by women in ophthalmology residency programmes, which they suggested could be due to trainers being more likely to give cases to male trainees. Female trainees also participated in fewer robotic colorectal procedures, with less operative time on the robotic console afforded [36]. Similarly, a systematic review highlighted female trainees in various specialties performed fewer cases per week and potentially had limited access to training facilities [37]. Eruchalu et al. [38] found that female trainees performed fewer cases, that is, until gender parity was reached, after which case logs were equivalent.

#### Technical skills

Antonoff et al. [39] found higher scores for men in coronary anastomosis skills, with women receiving more “fail” assessments. Dill-Macky et al. [40] analysed laparoscopic skill assessment using blinded videos of trainees and unblinded assessments. While there was no difference in blinded scores between genders, when comparing blinded and unblinded scores individually, assessors were less likely to agree on the scores of women compared to men. However, another study about laparoscopic skills by Skjold-Ødegaard et al. [41] reported higher performance scores in female residents, particularly when rated by women. The lowest score was shown in male trainees rated by men. While some studies showed disparities in assessment, several studies reported no difference in technical skill assessments (arthroscopic, knot tying, and suturing skills) between genders [42–46].

Several studies investigated trainees’ abilities to complete isolated tasks associated with surgical skills. In laparoscopic tasks, men were initially more skilful in peg transfer and intracorporeal knot tying than women. Following training, the performance was not different between genders [47]. A study on microsurgical skills reported better initial visual-spatial and perceptual ability in men, while women had better fine motor psychomotor ability. However, these differences were not significant, and all trainees improved significantly after training [48]. A study by Milam et al. [49] revealed men performed better in mental rotation tasks and women outperformed in working memory. They hypothesised that female trainees would experience stereotype threat, fear of being reduced to a stereotype, which would impair their performance. They found no evidence of stereotype threat influencing female performance, disproving their hypothesis, a finding supported by Myers et al. [50].

### Ethnicity and country of graduation

Most papers reported ethnicity and country of graduation concurrently, for example grouping trainees as White UK graduates (WUKG), Black and minority ethnicity UK graduates (BME UKG), and international medical graduates (IMG). Therefore, these areas will be addressed together in the following section.

#### Academic achievement

When assessing the likelihood of passing American Board of Surgery (ABS) examinations on first attempt, Yeo et al. [51] found that White trainees were more likely than non-White. They found that the influence of ethnicity was more significant in the end-of-training certifying exam than in the start-of-training qualifying exam. This finding was corroborated in a study of both the OITE and ABOS certifying exam, suggesting widening inequalities during training [52].

Two UK-based studies reported significantly higher MRCS pass rates in White trainees compared to BMEs [4, 14]. BMEs were less likely to pass MRCS Part A and B, though this was not true for Part A when variations in socioeconomic background were corrected for [14]. However, Robinson et al. [53] found no difference in MRCS pass rates based on ethnicity. Another study by Robinson et al. [15] demonstrated similar pass rates between WUKGs and BME UKGs, but IMGs had significantly lower pass rates than all UKGs. The FRCS pass rates of WUKGs, BME UKGs and IMGs were 76.9%, 52.9%, and 53.9%, respectively, though these percentages were not statistically significantly different [16].

There was no difference in MRCOG results based on ethnicity, but higher success rates were found in UKGs [19]. In FRCOphth, WUKGs had a pass rate of 70%, higher than other groups of trainees, with a pass rate of only 45% for White IMGs [52].

By gathering data from training programmes reporting little to no DA due to ethnicity, Roe et al. [54] were able to provide a list of factors they felt were protective against DA, such as having supportive supervisors and developing peer networks.

#### Assessment for progression

RCOphth [55] found higher rates of satisfactory ARCP outcomes for WUKGs compared to BME UKGs, followed by IMGs. RCOG [19] discovered higher rates of non-satisfactory ARCP outcomes from non-UK graduates, particularly amongst BMEs and those from the European Economic Area (EEA). Tiffin et al. [56] considered the difference in experience between UK graduates and UK nationals whose primary medical qualification was gained outside of the UK, and found that the latter were more likely to receive a non-satisfactory ARCP outcome, even when compared to non-UK nationals.

Woolf et al. [57] explored reasons behind DA by conducting interview studies with trainees. They investigated trainees’ perceptions of fairness in evaluation and found that trainees felt relationships developed with colleagues who gave feedback could affect ARCP results, and might be challenging for BME UKGs and IMGs who have less in common with their trainers.

#### Workplace-based assessment

Brooks et al. [58] surveyed the prevalence of microaggressions against Black orthopaedic surgeons during assessment and found 87% of participants experienced some level of racial discrimination during workplace-based performance feedback. Black women reported having more racially focused and devaluing statements from their seniors than men.

#### Surgical experience

Eruchalu et al. [38] found that white trainees performed more major surgical cases and more cases as a supervisor than did their BME counterparts.

### Technical skills

Dill-Macky et al. [40] reported no significant difference in laparoscopic surgery assessments between ethnicities.

### Individual and family background in education

#### Academic achievement

Two studies [4, 16] concentrated on educational background, considering factors such as parental occupation and attendance of a fee-paying school. MRCS part A pass rate was significantly higher for trainees for whom Medicine was their first Degree, those with university-educated parents, higher POLAR (Participation In Local Areas classification group) quintile, and those from fee-paying schools. Higher part B pass rate was associated with graduating from non-Graduate Entry Medicine programmes and parents with managerial or professional occupations [4]. Trainees with higher degrees were associated with an almost fivefold increase in FRCS success and seven times more scientific publications than their counterparts [16].

### Socioeconomic background

Two studies used Index of Multiple Deprivation quintile, the official measure of relative deprivation in England based on geographical areas for grading socioeconomic level. The area was defined at the time of medical school application. Deprivation quintiles (DQ) were calculated, ranging from DQ1 (most deprived) to DQ5 (least deprived) [4, 14].

#### Academic achievement

Trainees with history of less deprivation were associated with higher MRCS part A pass rate. More success in part B was associated with history of no requirement for income support and less deprived areas [4]. Trainees from DQ1 and DQ2 had lower pass rates and higher number of attempts to pass [14]. A general trend of better outcomes in examination was found from O&G trainees in less deprived quintiles [19].

#### Assessment for progression

Trainees from DQ1 and DQ2 received significantly more non-satisfactory ARCP outcomes (24.4%) than DQ4 and DQ5 (14.2%) [14].

### Age

#### Academic achievement

Trainees who graduated at age less than 29 years old were more likely to pass MRCS than their counterparts [4].

### Assessment for progression

Authors [18, 56] found that older trainees received more non-satisfactory ARCP outcomes. Likewise, there was higher percentage of non-satisfactory ARCP outcomes in O&G trainees aged over 45 compared with those aged 25–29 regardless of gender [19].

### Disability

#### Academic achievement

Trainees with disability had significantly lower pass rates in MRCS part A compared to candidates without disability. However, the difference was not significant for part B [59].

## Discussion

### What have we learnt from the literature?

It is heartening to note the recent increase in interest in DA (27 studies in the last 4 years, compared to 26 in the preceding 40) (Fig. 2). The vast majority (77%) of studies are quantitative, based in the US or UK (89%), focus on gender (85%) and relate to clinical assessments (51%) rather than examination results. Therefore, the surgical community has invested primarily in researching the experience of women in the USA and UK.

Interestingly, a report by RCOG [19] showed that men were more likely to receive non-satisfactory ARCP outcomes than women, and a study by Rushd et al. [17] found that women were more likely to pass part 2 of MRCOG than men. This may be because within O&G men are the “out-group” (a social group or category characterised by marginalisation or exclusion by the dominant cultural group) as 75% of O&G trainees are female [60].

This contrasts with other specialities in which men are the in-group and women are seen to underperform. Outside of O&G, in comparison to men, women are less likely to pass MRCS [4, 14], receive satisfactory ARCP outcome [16, 18], or receive positive feedback [24], whilst not performing the same number of procedures as men [34, 35]. This often leads to poor self-confidence in women [32], which can then worsen performance [21].

It proves difficult to comment on DA for many groups due to a lack of evidence. The current research suggests that being older, having a disability, graduate entry to medicine, low parental education, and living in a lower socioeconomic area at the time of entering medical school are all associated with lower MRCS pass rates. Being older and having a lower socioeconomic background are also associated with non-satisfactory ARCP outcomes, slowing progression through training.

These characteristics may provide a compounding negative effect – for example having a previous degree will automatically make a trainee older, and living in a lower socioeconomic area makes it more likely their parents will have a non-professional job and not hold a higher degree. When multiple protected characteristics interact to produce a compounded negative effect for a person, it is often referred to as “intersectional discrimination” or “intersectionality” [61]. This is a concept which remains underrepresented in the current literature.

The literature is not yet in agreement over the presence of DA due to ethnicity. There are many studies that report perceived discrimination, however the data for exam and clinical assessment outcomes is equivocal. This may be due to the fluctuating nature of in-groups and out-groups, and multiple intersecting characteristics. Despite this, the lived experience of BME surgeons should not be ignored and requires further investigation.

### What are the gaps in the literature?

The overwhelming majority of literature exploring DA addresses issues of gender, ethnicity or country of medical qualification. Whilst bias related to these characteristics is crucial to recognise, studies into other protected characteristics are few and far between. The only paper on disability reported striking differences in attainment between disabled and non-disabled registrars [59]. There has also been increased awareness about neurodiversity amongst doctors and yet an exploration into the experience of neurodiverse surgeons and their progress through training has yet to be published [62].

The implications of being LGBTQ + in surgical training have not been recognised nor formally addressed in the literature. Promisingly, the experiences of LGBTQ + medical students have been recognised at an undergraduate level, so one can hope that this will be translated into postgraduate education [63, 64]. While this is deeply entwined with experiences of gender discrimination, it is an important characteristic that the surgical community would benefit from addressing, along with disability. To a lesser extent, the effect of socioeconomic background and age have also been overlooked.

### Characterising trainees for the purpose of research

Ethnicity is deeply personal, self-defined, and may change over time as personal identity evolves, and therefore arbitrarily grouping diverse ethnic backgrounds is unlikely to capture an accurate representation of experiences. There are levels of discrimination even within minority groups; colourism in India means dark-skinned Indians will experience more discrimination than light-skinned Indians, even from those within in their own ethnic group [65]. Therefore, although the studies included in the scoping review accepted self-definitions of ethnicity, this is likely not enough to fully capture the nuances of bias and discrimination present in society. For example, Ellis et al. [4] grouped participants as “White”, “Mixed”, “Asian”, “Black” and “Other”, however they could have also assigned a skin tone value such as the NIS Skin Colour Scale [66], thus providing more detail.

Ethnicity is more than genetic heritage; it is also cultural expression. The experience of an IMG in UK postgraduate training will differ from that of a UKG, an Indian UKG who grew up in India, and an Indian UKG who grew up in the UK. These are important distinctions which are noted in the literature (e.g. by Woolf et al., 2016 [57]) however some do not distinguish between ethnicity and graduate status [15] and none delve into an individual’s cultural expression (e.g., clothing choice) and how this affects the perception of their assessors.

### Reasons for DA

Despite the recognition of inequalities in all specialties of surgery, there is a paucity of data explicitly addressing why DA occurs. Reasons behind the phenomenon must be explored to enable change and eliminate biases. Qualitative research is more attuned to capturing the complexities of DA through observation or interview-based studies. Currently most published data is quantitative, and relies on performance metrics to demonstrate the presence of DA while ignoring the causes. Promisingly, there are a gradually increasing number of qualitative, predominantly interview-based, studies (Fig. 2).

To create a map of DA in all its guises, an analysis of the themes reported to be contributory to its development is helpful. In our review of the literature, four themes have been identified:

#### Training culture

In higher surgical training, for there to be equality in outcomes, there needs to be equity in opportunities. Ellis et al. [4] recognised that variation in training experiences, such as accessibility of supportive peers and senior role models, can have implications on attainment. Trainees would benefit from targeted support at times of transition, such as induction or at examinations, and it may be that currently the needs of certain groups are being met before others, reinforcing differential attainment [4].

#### Experience of assessment

Most literature in DA relates to the presence (or lack of) an attainment gap in assessments, such as ARCP or MRCS. It is assumed that these assessments of trainee development are objective and free of bias, and indeed several authors have described a lack of bias in these high-stakes examinations (e.g., Ong et al., 2019 [12]; Robinson et al., 2019 [53]). However, in some populations, such as disabled trainees, there are differences in attainment [59]. This is demonstrated despite legislation requiring professional bodies to make reasonable adjustments to examinations for disabled candidates, such as additional time, text formatting amendments, or wheelchair-accessible venues [67]. Therefore it would be beneficial to investigate the implementation of these adjustments across higher surgical examinations and identify any deficits.

#### Social networks

Relationships between colleagues may influence DA in multiple ways. Several studies identified that a lack of a relatable and inspiring mentor may explain why female or BME doctors fail to excel in surgery [4, 55]. Certain groups may receive preferential treatment due to their perceived familiarity to seniors [35]. Robinson et al. [15] recognised that peer-to-peer relationships were also implicated in professional development, and the lack thereof could lead to poor learning outcomes. Therefore, a non-discriminatory culture and inclusion of trainees within the social network of training is posited as beneficial.

#### Personal characteristics

Finally, personal factors directly related to protected characteristics have been suggested as a cause of DA. For example, IMGs may perform worse in examinations due to language barriers, and those from disadvantaged backgrounds may have less opportunity to attend expensive courses [14, 16]. Although it is impossible to exclude these innate deficits from training, we may mitigate their influence by recognising their presence and providing solutions.

The causes of DA may also be grouped into three levels, as described by Regan de Bere et al. [68]: macro (the implications of high-level policy), meso (focusing on institutional or working environments) and micro (the influence of individual factors). This can intersect with the four themes identified above, as training culture can be enshrined at both an institutional and individual level, influencing decisions that relate to opportunities for trainees, or at a macro level, such as in the decisions made on nationwide recruitment processes. These three levels can be used to more deeply explore each of the four themes to enrich the discovery of causes of DA.

## Discussions outside of surgery

Authors in General Practice (e.g., Unwin et al., 2019 [69]; Pattinson et al., 2019 [70]), postgraduate medical training (e.g., Andrews, Chartash, and Hay, 2021 [71]), and undergraduate medical education (e.g., Yeates et al., 2017 [72]; Woolf et al., 2013 [73]) have published more extensively in the aetiology of DA. A study by Hope et al. [74] evaluating the bias present in MRCP exams used differential item functioning to identify individual questions which demonstrated an attainment gap between male and female and Caucasian and non-Caucasian medical trainees. Conclusions drawn about MRCP Part 1 examinations may be generalisable to MRCS Part A or FRCOphth Part 1: they are all multiple-choice examinations testing applied basic science and usually taken within the first few years of postgraduate training. Therefore it is advisable that differential item functioning should also be applied to these examinations. However, it is possible that findings in some subspecialities may not be generalisable to others, as training environments can vary profoundly. The RCOphth [55] reported that in 2021, 53% of ophthalmic trainees identified as male, whereas in Orthopaedics 85% identified as male, suggesting different training environments [5]. It is useful to identify commonalities of DA between surgical specialties and in the wider scope of medical training.

### Limitations of our paper

Firstly, whilst aiming to provide a review focussed on the experience of surgical trainees, four papers contained data about either non-surgical trainees or medical students. It is difficult to draw out the surgeons from this data and therefore it is possible that there are issues with generalisability. Furthermore, we did not consider the background of each paper’s authors, as their own lived experience of attainment gap could form the lens through which they commented on surgical education, colouring their interpretation. Despite intending to include as many protected characteristics as possible, inevitably there will be lived experiences missed. Lastly, the experience of surgical trainees outside of the English-speaking world were omitted. No studies were found that originated outside of Europe or North America and therefore the presence or characteristics of DA outside of this area cannot be assumed.

## Conclusion

Experiences of inequality in surgical assessment are prevalent in all surgical subspecialities. In order to further investigate DA, researchers should ensure all protected characteristics are considered - and how these interact - to gain insight into intersectionality. Given the paucity of current evidence, particular focus should be given to the implications of disability, and specifically neurodiversity, in progress through training as they are yet to be explored in depth. In defining protected characteristics, future authors should be explicit and should avoid generalisation of cultural backgrounds to allow authentic appreciation of attainment gap. Few authors have considered the driving forces between bias in assessment and DA, and therefore qualitative studies should be prioritised to uncover causes for and protective factors against DA. Once these influences have been identified, educational designers can develop new assessment methods that ensure equity across surgical trainees.

### Electronic supplementary material

Below is the link to the electronic supplementary material.

Supplementary Material 1

Supplementary Material 2

## Data Availability

All data provided during this study are included in the supplementary information files.

## Abbreviations

ACGME: Accreditation Council for Graduate Medical Education
ABOS: American Board of Orthopaedic Surgery
ABS: American Board of Surgery
ABSCE: American Board of Surgery Certifying Exam
ARCP: Annual Review of Competence Progression
BAME/BME: Black, Asian, and Minority Ethnicity
CREOG: Council on Resident Education in Obstetrics and Gynecology
DA: Differential Attainment
DQ: Deprivation Quintile
EEA: European Economic Area
EPA: Entrustable Professional Activities
FRCOphth: Fellowship of The Royal College of Ophthalmologists
FRCS: Fellow of the Royal College of Surgeons
GMC: General Medical Council
HST: Higher Surgical Training
IMG: International Medical Graduate
ITER: In-Training Evaluation Report
MRCOG: Member of the Royal College of Obstetricians and Gynaecologists
MRCP: Member of the Royal College of Physicians
MRCS: Member of the Royal College of Surgeons
O&G: Obstetrics and Gynaecology
OITE: Orthopaedic In-Training Examination
POLAR: Participation In Local Areas
PGY: Postgraduate Year
RCOphth: The Royal College of Ophthalmologists
RCOG: The Royal College of Obstetricians and Gynaecologists
RCS: The Royal College of Surgeons of England
UKG: United Kingdom Graduate
WUKG: White United Kingdom Graduate
