# Supplementary material for: Differential attainment in assessment of postgraduate surgical trainees: a scoping review
Source: BMC Med Educ. 2024 May 30;24:597. doi: 10.1186/s12909-024-05580-2 (PMC11141033; doi:10.1186/s12909-024-05580-2)
Supplement: Supplementary file 1 — Supplementary Material 1 [file 12909_2024_5580_MOESM1_ESM.docx]

Embase Classic+Embase <1947 to 2024 Week 17>

1 ethnicit*.mp. 199818

2 exp foreign medical graduate/ 470

3 international medical graduate*.mp. 1486

4 exp female physician/ 6315

5 female surgeon.mp. 138

6 gender.mp. 774641

7 exp sexual orientation/ 52159

8 LGBT*.mp. 8449

9 exp disadvantaged population/ 1340

10 disabilit*.mp. 462352

11 exp disabled person/ 60957

12 exp minority group/ 63464

13 socioeconomic factor*.mp. [mp=title, abstract, heading word, drug trade name, original title, device manufacturer, drug manufacturer, device trade name, keyword heading word, floating subheading word, candidate term word] 18629

14 1 or 2 or 3 or 4 or 5 or 6 or 7 or 8 or 9 or 10 or 11 or 12 or 13 1496431

15 surgical specialties.mp. 4894

16 exp surgeon/ 212196

17 special?ty train*.mp. 3283

18 surgical train*.mp. 33476

19 surgical resident*.mp. 4256

20 exp surgical registrar/ 146

21 15 or 16 or 17 or 18 or 19 or 20 243605

22 (differ* adj3 attainment).mp. [mp=title, abstract, heading word, drug trade name, original title, device manufacturer, drug manufacturer, device trade name, keyword heading word, floating subheading word, candidate term word] 792

23 (differ* adj3 outcome).mp. [mp=title, abstract, heading word, drug trade name, original title, device manufacturer, drug manufacturer, device trade name, keyword heading word, floating subheading word, candidate term word] 40234

24 (differ* adj3 achievement).mp. [mp=title, abstract, heading word, drug trade name, original title, device manufacturer, drug manufacturer, device trade name, keyword heading word, floating subheading word, candidate term word] 1215

25 (attainment adj3 gap).mp. [mp=title, abstract, heading word, drug trade name, original title, device manufacturer, drug manufacturer, device trade name, keyword heading word, floating subheading word, candidate term word] 69

26 disparit*.mp. 184447

27 exp prejudice/ 4466

28 exp gender bias/ 4562

29 exp implicit bias/ 982

30 22 or 23 or 24 or 25 or 26 or 27 or 28 or 29 234800

31 evaluation.mp. 2734765

32 assessment.mp. 3822325

33 exp clinical competence/ 69840

34 (clinical adj3 competenc*).mp. [mp=title, abstract, heading word, drug trade name, original title, device manufacturer, drug manufacturer, device trade name, keyword heading word, floating subheading word, candidate term word] 75083

35 performance.mp. 2184898

36 mrcs.mp. 682

37 frcs.mp. 1048

38 arcp.mp. 198

39 annual review of competency progression.mp. 30

40 rita.mp. 2173

41 record of in training assessment.mp. 12

42 revalidat*.mp. 2882

43 31 or 32 or 33 or 34 or 35 or 36 or 37 or 38 or 39 or 40 or 41 or 42 7679144

44 14 and 21 and 30 and 43 349
