# Supplementary material for: Differential attainment in assessment of postgraduate surgical trainees: a scoping review
Source: BMC Med Educ. 2024 May 30;24:597. doi: 10.1186/s12909-024-05580-2 (PMC11141033; doi:10.1186/s12909-024-05580-2)
Supplement: Supplementary file 2 — Supplementary Material 2 [file 12909_2024_5580_MOESM2_ESM.docx]

Supplemental table 1 – Relevant stakeholders whose websites were searched for grey literature

| United Kingdom and Ireland | |
| --- | --- |
|  | General Medical Council (GMC) |
|  | British Medical Association (BMA) |
|  | Royal College of Surgeons of England (RCS England) |
|  | Royal College of Surgeons of Edinburgh (RCSEd) |
|  | Royal College of Physicians and Surgeons of Glasgow (RCSPG) |
|  | Royal College of Ophthalmologists (RCOphth) |
|  | Royal College of Obstetricians and Gynaecologists (RCOG) |
|  | Royal College of Surgeons in Ireland (RCSI) |
|  | Irish College of Ophthalmologists (ICO) |
|  | Institute of Obstetricians and Gynaecologists at the Royal College of Physicians in Ireland (RCPI) |
| North America | |
|  | American College of Surgeons (ACS) |
|  | American Academy of Orthopaedic Surgeons (AAOS) |
|  | American Academy of Ophthalmology (AAO) |
|  | American Academy of Otolaryngology - Head and Neck Surgery (AAO-HNSF) |
|  | The American College of Obstetricians and Gynecologists (ACOG) |
|  | The American Board of Surgery (ABS) |
|  | American Board of Medical Specialties (ABMS) |
|  | The American Society of Breast Surgeons (ASBrS) |
|  | Royal College of Physicians and Surgeons of Canada |
|  | Canadian Medical Association (CMA) |
|  | Canadian Association of General Surgeons (CAGS) |
|  | Canadian Ophthalmological Society |
|  | Canadian Orthopaedic Association (COA) |
| Germany | |
|  | German Society of Surgery (DGCH) |
|  | German Ophthalmology Society (DOG) |
|  | German Society of Oto-Rhino-Laryngology, Head and Neck Surgery (HNO) |
| Scandinavia | |
|  | Scandinavian Surgical Society (SSS/NKF) |
|  | Nordic Federation of Societies of Obstetrics and Gynecology (NCOG) |
|  | Nordic Orthopaedic Federation (NOF) |
|  | Nordic Association of Oto-Rhino-Laryngology, Head and Neck Surgery (ORL-HNS) |
| Australasia | |
|  | Royal Australasian College of Surgeons (RACS) |
